# A coronary artery fistula having connection between 2 coronary arteries and the left ventricle: A case report

**DOI:** 10.1097/MD.0000000000008546

**Published:** 2017-11-10

**Authors:** Chang-Yeon Kim, Ji Yong Choi, Kee Sik Kim

**Affiliations:** Division of Cardiology, Daegu Catholic University Medical Center, Daegu, Korea.

**Keywords:** dyspnea, fistula, heart ventricles

## Abstract

**Rationale:**

Cases of coronary artery fistula having a connection with the cardiac cavity are rare. Here, we report a case in which 2 coronary arteries empty into the left ventricular cavity together.

**Patient concerns:**

A 63-year-old woman who was diagnosed as having hypertension 20 years prior presented with dyspnea.

**Diagnoses:**

The coronary angiography revealed coronary artery fistula.

**Interventions:**

Chest X-ray showed pulmonary edema. Transthoracic echocardiography revealed moderately decreased left ventricular (LV) function and increased LV end-diastolic volume and mass index. Coronary angiography and cardiac computed tomography revealed that 2 coronary arteries joined together at the distal end and directly drained into the left ventricular cavity bypassing the myocardial capillary vessels. We started medical treatment for heart failure with an angiotensin-converting-enzyme inhibitor, loop diuretic, and spironolactone.

**Outcomes:**

The pulmonary edema improved rapidly. The patient did not experience dyspnea after discharge, and follow-up echocardiography showed improved cardiac function.

**Main lesson:**

Coronary artery fistula could be found incidentally on coronary angiography performed for varied reasons. Physicians must decide carefully whether the fistula needs to be treated in view of the clinical context.

## Abstract 2

Supplemental Digital Content is available in the text

## 1 Introduction

The coronary arteries originate from the aortic sinuses and progressively divide into smaller branches and then the microvascular bed, supplying blood to the myocardium. The capillary blood flow drains into the venules, which merge to form cardiac veins, and finally, the coronary sinus, which empties into the right atrium. Coronary artery fistula (CAF) is a type of coronary artery anomaly in which there is a direct connection between one or more coronary arteries and any of the 4 chambers or any of the great vessels. The exact incidence of CAF is unknown. The majority of the fistulas drain into the right heart system,^[1]^ and most patients (∼95%) with CAF have a single fistula.^[2]^ CAF having communication with 1 of the 4 cardiac chambers is called cameral fistula, which is rare, and presents in approximately 0.08% to 0.3% of patients undergoing coronary angiography. Most (∼90%) cameral fistulas drain into the right ventricle.^[3–5]^ Here, we report a rare case of cameral fistula in which a left and a right coronary artery joined together and then drained into the left ventricle.

## 2 Case description

A 63-year-old female patient presented with acute dyspnea to the emergency department. She said she had been having mild dyspnea for 6 months, but did not visit a doctor because the symptom occurred intermittently. Her initial vital signs were blood pressure (BP) of 200/100 mm Hg, heart rate of 124 beats/min, respiratory rate of 23/min, and oxygen saturation of 90%. An initial chest X-ray revealed pulmonary edema and mild cardiomegaly with a cardiothoracic (CT) ratio of 54%. Laboratory testing revealed a slightly elevated white blood cell count of 10,800/μL, and hemoglobin and C-reactive protein levels were within the normal range. The level of pro-brain natriuretic peptide was elevated at 1326 (21.4–189.2) pg/mL. Cardiac enzymes were within normal limits. She had been diagnosed with hypertension 20 years prior, but she had not taken hypertensive medication for the last 10 years. Her systolic BP decreased to 130 to 150 mm Hg several hours later, without specific medication. Electrocardiography showed sinus tachycardia of 124 beats/min and inverted T-waves in the precordial leads. Transthoracic echocardiography showed moderately reduced left ventricular (LV) ejection fraction (37% by Simpson's biplane method; Supplementary video 1) with LV enlargement (LV end-diastolic dimension of 6 cm). Septal and LV posterior wall thicknesses were 9.7 to 10.2 mm, and LV end-diastolic volume (EDV) increased (130 mL by biplane disc-summation method, 80 mL/m^2^). The left ventricular mass index (LVMI) was also elevated to 190 g/m^2^. The average global longitudinal strain was −8.6%. There was no evidence to suspect valvular heart disease.

**Figure 1:**
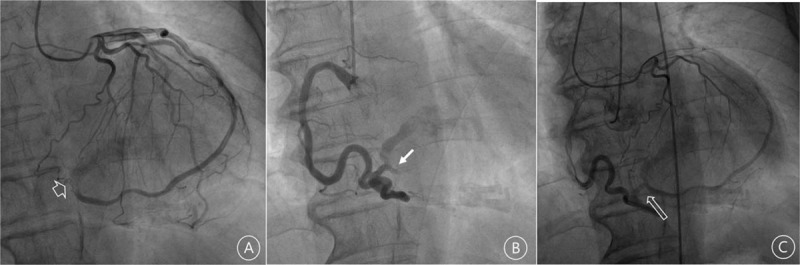
(A) Left coronary angiography showing the course of the intermedius artery. The short arrow indicates the entering point. (B) Right coronary angiography showing tortuous RCA emptying into a certain cavity, and another artery (filled arrow) near the entering point. (C) With simultaneous contrast injection, it was demonstrated the intermedius and RCA joined together (long arrow) and emptied into the cavity. RCA = right coronary artery.

Pulmonary edema improved rapidly with loop diuretic. Coronary angiography was performed to identify ischemic heart disease. The angiography revealed abnormal courses of anomalous coronary arteries, but no significant stenosis in epicardial coronary artery. The ramus intermedius artery did not taper along the course at all, and drained into a certain cavity (Fig. 1A). The right coronary angiography also showed the same pattern as the intermedius artery (Fig. 1B). After dual coronary injection, we identified that the 2 arteries joined near the termination point and seemed to drain together into the LV cavity (Fig. 1C; Supplementary video 2).

**Figure 2:**
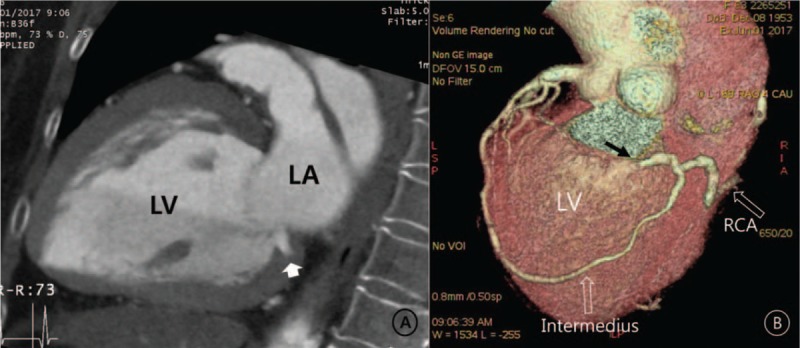
The cardiac computed tomography proved the fistula (short arrow) penetrated the basal inferior wall of the LV (A). The 3-dimensional volume-rendering image clearly displayed the courses of the intermedius artery and RCA. A black arrow: entering point (B). LV = left ventricle, RCA = right coronary artery.

**Figure 3:**
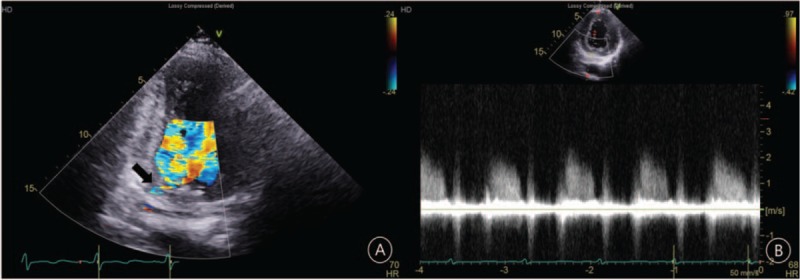
The modified apical 2-chamber view showing the shunt flow (black arrow) into the left ventricle (A). Continuous-wave Doppler recording of the shunt flow (B).

We performed cardiac computed tomography (CCT) and focused echocardiography to check the exact entering point. CCT showed a clear-cut view of the anomalous coronary artery course and the entering point, the basal LV (Fig. 2). Echocardiography showed a small defect in the basal inferior wall and an abnormal blood flow through the defect, occurring only during diastole (Fig. 3A; Supplementary video 3). The peak flow velocity in early diastole was about 2 m/s (Fig. 3B). Finally, the patient was diagnosed with a CAF with connection between the right coronary artery, intermedius artery, and the LV cavity. The amount of shunt seemed to be too small to cause heart failure. We chose to administer medical treatment with angiotensin-converting-enzyme inhibitor (ramipril 2.5 mg once a day), loop diuretic (furosemide 20 mg twice a day), and spironolactone (12.5 mg once a day). After discharge, her BP was tolerable, the congestion had completely disappeared, and her CT ratio had decreased to 48%. A low dose beta-blocker (bisoprolol 0.625 mg once a day) was added to her medication regimen. Four months later, LV ejection fraction improved to 51% (Supplementary video 4) and also the average global longitudinal strain to −12.2%. LV end-diastolic dimension decreased to 5.2 cm, and LVEDV was normalized to 86 mL (54 mL/m^2^).

## 3 Discussion

Most CAFs do not cause any symptoms and are, instead, often discovered incidentally on diagnostic coronary angiography or during a work-up of cardiac murmur.^[4]^ CAFs may give rise to congestive heart failure, arrhythmia, myocardial ischemia, and infective endocarditis. Because the causal relationship between the CAF and the patient's condition is often obscure, there is no consensus regarding the optimal management of a CAF. Conservative medical management is generally accepted as adequate in most cases of CAF. The treatment options in cases of hemodynamically significant CAF are surgical ligation and catheter closure. There are no standard methods to evaluate the functional importance of CAFs. Cardiac catheterization can be used in the quantification of left-to-right shunts,^[6]^ and there are several reports in which myocardial perfusion single-photon emission computed tomography or fractional flow reserve was used to assess myocardial ischemia.^[7,8]^

There have been several case reports about cameral fistulas that were found incidentally on echocardiography.^[9–11]^ They were big enough to be noticed at a glance. However, the patients in these reports exhibited normal cardiac functions and no notable cardiac symptoms. Therefore, the treatment option was to observe.

Our patient's echocardiography revealed moderately decreased systolic function, and the shunt was small, so we did not recognize it at the time of first examination. After coronary angiography, we were able to find the tiny shunt flow at LV basal segment, which occurred only in diastole. Our case was a little complicated due to the patient's heart function. She might have hypertensive heart disease at the time of presentation because, despite being diagnosed with hypertension 20 years ago, she had not taken any medicine for the last 10 years. LV wall thickness increased, and LVMI was markedly elevated. Alternatively, she could have had idiopathic dilated cardiomyopathy because during admission her systolic BP ranged between 110 and 150 mm Hg without any administration of antihypertensive medication. The CAF seemed to be an incidental finding and not the cause of the heart failure because the shunt was too small to bring about hemodynamic impact, and echocardiography showed no regional wall motion abnormalities. Although her left anterior descending artery (LAD) and left circumflex artery (LCX) were not related to the CAF and had no significant stenosis, the function of the myocardium supplied by the LAD and LCX also decreased. She was treated with optimal medical treatment for heart failure. She did not report any heart failure symptoms in the outpatient clinic.

CAFs could be found sometimes on coronary angiography performed for varied reasons. Physicians must decide carefully whether the CAF, once identified, needs to be treated, taking various clinical factors into consideration, including the patient's history, symptoms, echocardiographic data, and the hemodynamic impact of the CAF.

## Supplementary Material

Supplemental Digital Content

## Supplementary Material

Supplemental Digital Content

## Supplementary Material

Supplemental Digital Content

## Supplementary Material

Supplemental Digital Content
